# Transjugular intrahepatic portosystemic shunt using the FLUENCY expanded polytetrafluoroethylene-covered stent

**DOI:** 10.3892/etm.2012.776

**Published:** 2012-10-30

**Authors:** QINGHUA WU, JIANWEI JIANG, YUJIE HE, TIANPENG JIANG, SHI ZHOU

**Affiliations:** 1 Department of Radiology, The Third Affiliated Hospital of Nantong University;; 2 Department of Radiology, The Fourth Hospital of Wuxi, Wuxi 214042;; 3 Department of Interventional Radiology, The Affiliated Hospital of Guiyang Medical College, Guiyang, Guizhou 550004, P.R. China

**Keywords:** portosystemic shunt, interventional radiography, portal hypertension, endovascular stent

## Abstract

The aim of this study was to evaluate the feasibility of transjugular intrahepatic portosystemic shunts using FLUENCY expanded polytetraf luoroethylene (PTFE)-covered stents and the effect on the patency rate. A total of 114 cirrhotic patients who were treated by transjugular intrahepatic portosystemic stent shunt (TIPS) placement using a FLUENCY expanded, PTFE-covered stent were enrolled in the present study. Of the patients, 15 underwent an additional bare metal stent implantation on the portal side of the covered stent, simultaneously. Patients underwent Doppler sonography during the follow-up. Mean portal venous pressure dropped from 2.499±0.588 cmHg to 1.764±0.294 cmHg. The cumulative patency rates for one and two years were 86.7% and 75.2%, respectively. The results demonstrate the feasibility of TIPS placement with the FLUENCY expanded PTFE-covered stent. TIPS placement with the FLUENCY expanded PTFE-covered stent was able to improve patency when compared with the use of conventional bare-metal stents.

## Introduction

Transjugular intrahepatic portosystemic stent shunt (TIPS) is an effective treatment for esophageal variceal hemorrhage caused by portal hypertension (1,2). It has been demonstrated that compared with medical treatment and endoscopic treatment, the early use of a TIPS in patients with hemorrhages improves the survival rate significantly. However, the TIPS technique was previously difficult to apply due to the low long-term patency rate of the bare metal stents and the cost of re-intervention (3–5). An estimated 80% of the world’s TIPS are now created with Viatorr stent grafts and numerous retrospective, prospective and randomized trials comparing their efficacy with those of bare stents have identified that long-term patency rates have increased significantly (6–9). however, it remains necessary to investigate new and more convenient stent grafts. Studies have revealed that stent thrombosis, pseudointimal hyperplasia, ingrowth of liver tissue into the shunt and hemodynamic disorders caused by stent angulations result in postoperative shunt stenosis and obstruction (10). Theoretically, the structure of a covered stent such as the FLUENCY expanded, polytetrafluoroethylene (PTFE)-covered stent may avoid stent dysfunction, including bile leakage, thrombosis and abnormal pseudointimal proliferation (11–14). Although the use of covered stents in TIPS has been rare, it is becoming increasingly valued. Therefore the efficacy of FLUENCY expanded, PTFE-covered stents was explored in the present study.

## Materials and methods

### General materials

The FLUENCY PTFE-coated self-expanding nitinol stent manufactured by BARD (Murray Hill, NJ, USA) was used for 114 patients in TIPS surgery, including 77 males and 37 females with an average age of 54±14 years. The patients all suffered from hepatic cirrhosis decompensation with portal hypertension. There were 92 cases of pure esophageal variceal disruption hemorrhage, 8 of pure refractory cirrhotic ascites and 14 of esophageal variceal disruption hemorrhage with refractory ascites. According to the Child-Pugh Liver function class, there were 29 cases of class A liver function, 68 of class B and 34 of class C. Written and informed consent was obtained from every patient and the study was approved by the ethics review board of Guiyang Medical College (Guiyang, China).

### Preoperative preparation

Cardiopulmonary, liver and coagulation functions were analyzed prior to TIPS to exclude surgical contraindications. Hypoproteinemia and coagulation disorders were corrected. Enhanced CT scans of the abdomen and other images were examined to observe the location and anatomical associations of the portal vein and its branches, while excluding the thrombosis of the portal vein and inferior vena cava and portal vein cavernous transformation. In cases without enhanced CT scans, indirect portal venography was performed prior to the TIPS procedure.

### Operative technique

The right-internal jugular vein was selected and the right or left branch was punctured through the right-hepatic or hepatic vein in order to measure the portosystemic pressure gradient (PSG) and select a balloon catheter to expand the portosystemic shunt. When the PSG decreased to <1.176 cmHg, the covered stent was implanted in accordance with the corresponding diameter. The parenchymal section and the hepatic vein side of the shunt required complete coverage of the stents. The varicose gastric coronary vein was embolized intraoperatively. Stents with a diameter of 8 mm and length of 60 mm were implanted. Bare metal stents were implanted into 15 patients and covered stents of the same diameter were also implanted simultaneously in the hepatic vein side.

### Postoperative treatment

The patients were required to avoid dietary protein for 2 weeks to maintain smooth stools. Low-molecular weight heparin was administered via subcutaneous injection according to the coagulation conditions. Antiplatelet therapy with 75 mg clopidogrel was administered orally each day. Coagulation was monitored during the medication period.

### Follow-up

During the follow-up, recurrent bleeding, ascites and complications were observed. At 7 days, 1, 3 and 6 months and 1 year following TIPS implantation, Doppler ultrasound was performed to examine the shunt. After that time, liver Doppler ultrasound examination was performed every 6 months. Follow-up lasted until March 30, 2012. When recurrent gastrointestinal bleeding occurred or ultrasound examination revealed shunt dysfunction, portography was performed and intervention applied with the use of balloon dilation and bare metal stent implantation to support the narrow shunt. Loss of follow-up, mortality and the emergence of shunt dysfunction were all classified as follow-up termination.

### Statistical analysis

Shunt patency rates were assessed using a Kaplan-Meier survival curve. The portal venous pressure and mean pressure gradient were assessed with the paired t-test. P<0.01 was considered to indicate a statistically significant difference.

## Results

### Clinical efficacy

**Figure 1:**
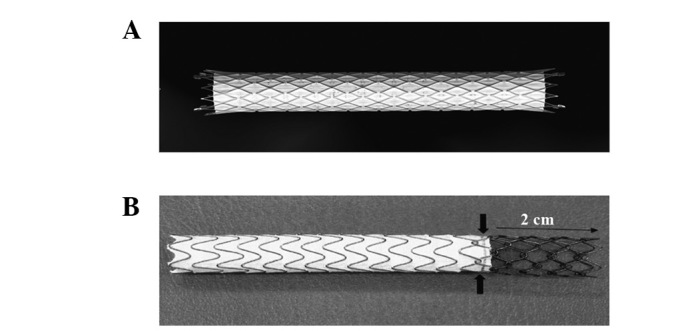
(A) FLUENCY fully-covered stent with fully-covered structure and 2 mm of exposed section at each end. (B) TIPS stent with 2-cm long bare metal stent in the portal vein side. Arrows indicate the boundary between the coated and bare sections of the stent. TIPS, transjugular intrahepatic portosystemic stent shunt.

All patients achieved a 100% technical success. The covered stents (Fig. 1A) in the 114 cases were 8 mm in diameter. Bare metal stents of the same diameter were implanted in 15 cases simultaneously. The mean portal venous pressure dropped from 2.499±0.588 cmHg to 1.764±0.294 cmHg.

The varicose gastric coronary vein was also embolized in the procedure so that 37 of the 40 patients stopped bleeding within 24 h after emergency surgery. Seven days after surgery, three patients succumbed to various causes: one patient with alcoholic cirrhosis had continuous heavy bleeding 24 h after surgery and succumbed to disseminated intravascular coagulation (DIC) and multiple organ failure within 48 h; one patient stopped bleeding but succumbed to multiple organ failure on the third day after the surgery; and one patient had intraoperative splenic vein occlusion so that the coronary vein embolization could not be performed. The patient had 24 h of bleeding, and succumbed to DIC and multiple organ failure within 72 h. Eight cases of pure refractory ascites subsided significantly after two days. Two of the 14 patients with gastrointestinal bleeding and massive ascites succumbed after one week. Of the remaining 12 cases, ascites were significantly reduced in eight cases after two weeks and ascites were reduced slightly in one other case.

One patient with pure refractory ascites exhibited symptoms of hypovolemic shock 3 h after surgery with significant abdominal distension. Diagnostic peritoneal puncture revealed a bloody fluid that was not solidified. Laparotomy surgery was performed 4 h after TIPS and revealed a petechia on the top right of the hepatoduodenal ligament with a visible break of 2-mm diameter. Varicose veins on the right side of the hepatic portal were ruptured and bleeding and ascites subsided after one month of treatment. Besides this, operation-associated complications were not observed. Hepatic encephalopathy occurred in 23 patients after one month, of which two cases were stage IV and improved following medical treatment, while the remaining 21 cases exhibited stage I or II hepatic encephalopathy, dizziness, drowsiness, confusion and abnormal behaviors. All the symptoms disappeared following medical treatment.

With the exception of cases of lost follow-up and mortality from of other causes, 19 cases of recurrent bleeding were observed between postoperative days 3 and 1597, and three patients succumbed within seven days. A further 16 cases were confirmed as shunt occlusion or stenosis using liver Doppler ultrasound or portal venography. Of these 16 cases, five were thrombosis due to stenosis of the hepatic vein, three were thrombosis caused by portal vein blockage due to the cover of the stent and eight were thrombosis due to the cover of the stent overlay on the portal vein wall, resulting from the small angle between the stent portal end and portal vein wall. In two of the 16 patients, TIPS was performed again in the left branch of the portal vein via hepatic vein puncture, while in 12 cases the occluded portal vein was opened and expanded by a shunt balloon, with a bare metal stent implanted temporarily to support the hepatic or portal vein side. The remaining two cases failed to be treated due to widely occurring thrombosis in the portal vein.

### Shunt patency

**Figure 2:**
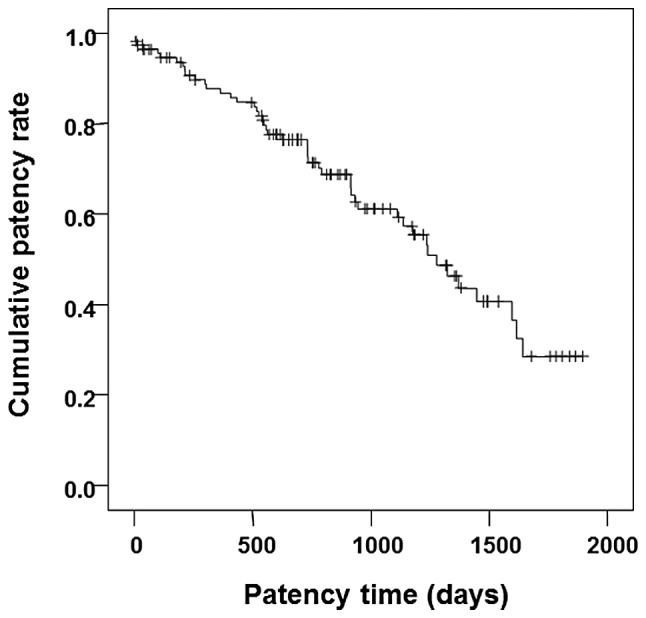
Shunt patency curve. The one-year cumulative patency rate was 86.7% and the two-year patency rate was 75.2%.

In the present study, the one-year cumulative patency rate was 86.7% and the two-year patency rate was 75.2% (Fig. 2). These were slightly higher than the patency rate of the dedicated TIPS Viatorr stent reported previously (15) and significantly higher than previously reported bare-metal stent patency rates (16–17). There was a significant difference between the covered and bare stents but no significant difference was observed between the covered and Viatorr stents.

## Discussion

In the present study, the one-year cumulative patency rate was slightly higher than the patency rates of dedicated TIPS stents revealed in previous studies and was significantly higher than previously reported bare metal stent patency rates (15–17). The covered stent was almost entirely covered with a PTFE membrane, leaving exposed sections only at each end with lengths of 2 mm (Fig. 1A), in order to facilitate the placement of tantalum markers. However, the dedicated Viatorr stent has a 2 cm uncovered section on the portal vein side, which is separated by metal rings and a gold marker (Fig. 1B). Due to the great tissue compatibility of PTFE, it did not stimulate thrombosis, prevented bile leakage and provided a good matrix for neointimal coverage of the stent surface. Thus the rate of shunt patency was improved.

The treatment of bleeding in the present study was also satisfactory, with 37 out of 40 cases of acute bleeding being effectively controlled. However the occurrence of hepatic encephalopathy was not avoided or resolved by the TIPS technique. On the basis of effectively reducing the portal pressure and PSG, a small-diameter stent would theoretically reduce the occurrence and extent of hepatic encephalopathy. Compared with the bare metal stents reported previously, although the patency rate in the present study was improved, the incidence of hepatic encephalopathy was not reduced. Early in the present study, a 10-mm stent was adopted to control the portal vein pressure. Afterwards 8-mm stents were selected since a reduction of the shunt volume may theoretically control the incidence of hepatic encephalopathy. However, a comparative study of the incidences of hepatic encephalopathy between various diameter shunts was not performed due to the limited number of cases. Saxon *et al*(18) considered the 8-mm stent to be the best choice for both the prevention of hepatic encephalopathy and appropriate diversion but it is unknown whether it is suitable for the physiological characteristics of Asian populations or hepatitic cirrhosis. Whether ≤7-mm stents are more suitable in Asian populations for reducing the incidence of hepatic encephalopathy while maintaining a stable shunt volume, requires larger samples of clinical case observations and studies.

In practical applications, fully-covered FLUENCY stents have the following two benefits: i) simplified surgical procedures; and ii) fully-covered structures which reduce the incidence of portal vein hemodynamic disorder. However, fully-covered structures and the woven metal structure also have certain problems. Firstly, when the stent is too deep in the portal vein, the stent cover may overlay the portal vein which may lead to portal vein thrombosis and shunt dysfunction. Three cases of obstruction of the portal vein branch occurred in the present study, resulting in significant increases in PSG. The Viatorr stent is effective for avoiding this situation and the structure of bare metal stents in the portal vein side enables the covered section to maintain a certain distance, thus preventing the ‘cap’ and reducing the risk of cover obstruction of the portal vein branch. Therefore, further study of the association between two stents is necessary.
